# Epigallocatechin-3-Gallate attenuates lipopolysacharide-induced pneumonia via modification of inflammation, oxidative stress, apoptosis, and autophagy

**DOI:** 10.1186/s12906-024-04436-y

**Published:** 2024-04-05

**Authors:** Meili Shen, Yuting You, Chengna Xu, Zhixu Chen

**Affiliations:** 1 Pediatric Critical Care Medicine Department, Quanzhou Children’s Hospital (Quanzhou Maternal and Child Health Hospital), Fengze District, Quanzhou City, Fujian Province 362000 China; 2 Children’s Respiratory Department, Quanzhou Children’s Hospital (Quanzhou Maternal and Child Health Hospital), Fengze District, Quanzhou City, Fujian Province 362000 China

**Keywords:** Pneumonia, Apoptosis, Autophagy, Inflammation, Oxidative stress, Therapy

## Abstract

**Background:**

Pneumonia, the acute inflammation of lung tissue, is multi-factorial in etiology. Hence, continuous studies are conducted to determine the mechanisms involved in the progression of the disease and subsequently suggest effective treatment. The present study attempted to evaluate the effects of Epigallocatechin-3-Gallate (EGCG), an herbal antioxidant, on inflammation, oxidative stress, apoptosis, and autophagy in a rat pneumonia model.

**Methods:**

Forty male Wistar rats, 5 months old and 250–290 g were divided into four groups including control, EGCG, experimental pneumonia (i/p LPS injection, 1 mg/kg), and experimental pneumonia treated with EGCG (i/p, 15 mg/kg, 1 h before and 3 h after LPS instillation). Total cell number in the bronchoalveolar lavage fluid, inflammation (TNF-a, Il-6, IL-1β, and NO), oxidative stress (Nrf2, HO-1, SOD, CAT, GSH, GPX, MDA, and TAC), apoptosis (BCL-2, BAX, CASP-3 and CASP-9), and autophagy (mTOR, LC3, BECN1) were evaluated.

**Results:**

The findings demonstrated that EGCG suppresses the LPS-induced activation of inflammatory pathways by a significant reduction of inflammatory markers (*p*-value < 0.001). In addition, the upregulation of BCL-2 and downregulation of BAX and caspases revealed that EGCG suppressed LPS-induced apoptosis. Furthermore, ECGC suppressed oxidative injury while promoting autophagy in rats with pneumonia (*p*-value < 0.05).

**Conclusion:**

The current study revealed that EGCG could suppress inflammation, oxidative stress, apoptosis, and promote autophagy in experimental pneumonia models of rats suggesting promising therapeutical properties of this compound to be used in pneumonia management.

## Background

Pneumonia is described as a prevalent infectious disease, characterized by the infection of the pulmonary parenchyma caused by a variety of pathogens such as bacteria, viruses, and fungi [1]. It is recently considered that the morbidity and mortality rates due to pneumonia are rising all over the world [2, 3]. Importantly, it is established that pneumonia is the leading cause of death in children aged < 5 years, as annually the disease leads to approximately 1.3 million deaths of children globally [4]. Unfortunately, pneumonia caused by drug-resistant species and the inefficiency of current treatment strategies [4], as well as the lack of clarity about the underlying molecular mechanisms of lung injury after infection, are among the challenges of pneumonia management in current clinics. Therefore, extensive research has been conducted to elucidate the mechanisms involved in tissue damage caused by pneumonia and to propose a novel treatment approach.

Alterations in pathways involved in regulated cell death including apoptosis, the most well-known type of programmed cell death, and autophagy, an evolutionarily conserved cellular pathway that recycles unnecessary cytoplasmic materials, may contribute to the exacerbation of pneumonia-induced lung injury [5]. Moreover, the intensification of immune responses and increased secretion of immune mediators along with the presence of immune cells, in addition to confronting pathogens, may cause undesired damage to the respiratory system [6]. Disturbance of the oxidative balance through overproduction of free radicals and/or inhibition of antioxidant defenses may synergize with the exacerbation of inflammation and cell death [7, 8]. Therefore, clarifying the exact role of the mentioned pathways in the lung tissue damaged by pneumonia and subsequently proposing a treatment strategy that functions through the modulation of these mechanisms can be of critical clinical importance.

The administration of herbal antioxidants, which, in addition to the ability to modulate the mentioned pathways, represent high pharmaceutical safety and considerable antiinfection properties, have been suggested as novel strategies by a variety of studies [9–13]. Epigallocatechin-3-gallate (EGCG) is considered one of the main components of green tea with modulatory properties on inflammation, cell death, and lung injury. Cumulative studies demonstrate that EGCG prevents inflammation and augments antioxidant defense [14, 15]. Moreover, EGCG has the ability to regulate fundamental molecular pathways contributing to cell survival, homeostasis, proliferation, and death [16, 17]. Interestingly, a recent study suggested the potential of EGCG to attenuate acute lung injury via the regulation of macrophage polarization and immune responses. It has been demonstrated that EGCG is able to alleviate LPS-induced acute lung injury and inflammatory response by increasing the expression of PRKCA [18]. Also, the antimicrobial properties of EGCG have been suggested in several studies, which may propose EGCG as one of the new strategies to deal with pulmonary complications, especially pneumonia [19–21]. Nevertheless, the effect of EGCG on autophagy, apoptosis, inflammation, and oxidative stress and as a result, amelioration of pneumonia-induced lung injury is not clarified.

Taken together, pneumonia is considered one of the main health concerns worldwide, and its treatment faces remaining challenges. As a result, continuous investigations sought to suggest novel therapeutic strategies. Therefore, the present study aimed to assess the effects of EGCG on markers of apoptosis, autophagy, inflammation, and oxidative stress in an animal model of lipopolysaccharide (LPS)-induced pneumonia.

## Materials and methods

### Ethical approval

The current animal study was designed and conducted according to the National Institutes of Health Laboratory Animal Care and Use Guidelines. Ethical Committee of Quanzhou Children’s Hospital approved this study (Number 107–2023, 2023.11.7).

### Animals and study design

A total of 40 male *Wistar rats*, 5 months old and weighing approximately 250–290 g were provided by the Institutional Animal Care and Use Committee. Animals were acclimated to the laboratory conditions for three weeks and the health condition of all rats, for starting the experiment, was checked and approved by a local specialist at the Institutional Animal Care and Use Committee. The rats were randomly divided into the following four groups where each group contained 10 animals: control (CON) group (received sterile saline 0.9% ip [the vehicle for both LPS and EGCG]), EGCG (i/p, 15 mg/kg, 1 h before and 3 h after LPS instillation) group, experimental pneumonia (i/p LPS injection, 1 mg/kg) group, and LPS + EGCG group. EGCG was purchased from BioCrick Biotech (Chengdu, Sichuan Province, China) and intraperitoneally administered to rats. The doses of LPS and EGCG were selected according to similar studies published previously [19, 22]. Animals were sacrificed 24 h after pneumonia induction under ketamine 10% (BREMER PHARMA GMBH, 34,414 Warburg, Germany) and xylazine 2% (Alfasan, Woerden, Holland) anesthesia (injected IM) and tissue samples were collected. Lungs were subjected to bronchoalveolar lavage fluid (BALF) collection [23].

### Lung wet-to-dry weight ratio measurement

The isolated lung samples were weighed immediately. Then, the samples were dried until the stabilization of weight. Finally, the wet-to-dry weight (W/D) ratio was obtained.

### Cell number in BALF

The isolated BALF samples were centrifuged at 1500 g for 12 min at 4 °C, cell pellets were dissolved in saline, and cell numbers in the suspension were measured with an automatic blood cell counter (Sysmex E-25,000; Toua-iyoudenshi Co. Ltd, Japan).

### Tissue RNA isolation and cDNA synthesis

Total RNA was isolated from lung tissues using the Trizol reagent (Sigma-Aldrich, Kenilworth, USA, Cat: T9424) according to the manufacturer’s protocol. The integrity and purity of isolated RNA were determined using 1/5% agarose gel electrophoresis and NanoDrop Spectrophotometer ND1000 (NanoDrop Technologies Inc, USA), respectively. Reverse transcription was performed using a First Strand cDNA Synthesis Kit (ThermoFisher, Cat: K1621). Synthesized single-stranded DNA was stored at -20 °C for further analysis.

### Real-time quantified polymerase chain reaction

The levels of gene expression were analyzed using real-time quantified chain reaction (RT-qPCR). To perform RT-qPCR specific primers were designed (Table 1) and the expression levels of genes related to inflammation (tumor necrosis factor-alpha [TNF-α], interleukin [IL]-6, IL-1b, nitric oxide [NO]), oxidative stress (nuclear factor erythroid-derived 2-like 2 [NFE2L2], heme oxygenase 1 [HO-1]), apoptosis (B-cell lymphoma 2 [BCL-2], BCL-2-like protein 4 [BAX], Caspase [CASP]-3, and CASP-9), and autophagy (mammalian target of rapamycin [mTOR], microtubule-associated protein 1 A/1B-light chain 3 [LC3], beclin-1 [BECN1]) in lung tissue were detected. β-actin was considered as the internal control and A SYBR Green and a real-time PCR system (7500 system, Applied Biosystems, Carlsbad, California, USA) were used. In order to confirm that only a single amplified PCR product was assessed the melting curve was constructed. Samples were analyzed in triplicate by the well-known 2^−ΔΔCT^ method. In this regard, standard deviations (SD) of threshold cycle [24] values not exceeding 0.5 on a within-run basis were included [23].

**Table 1:** The primers used for RT-qPCR assay

Genes	Primers	Sequences (5’→3’)
***BCL-2***	Forward	CTTTGAGTTCGGAGGGGTCA
	Reverse	AAATCAAACAGGGGCCGCAT
***BAX***	Forward	GCCCTTTTGCTTCAGGGTTT
	Reverse	ACAGCTGCGATCATCCTCTG
***CASP-3***	Forward	CTGAGGGTCAGCTCCTAGCG
	Reverse	CCAGAGTCCATTGATTTGCTTC
***CASP-9***	Forward	GATCAGGCCAGGCAGCTAAT
	Reverse	CGGCTTTGATGGGTCATCCT
***mTOR***	Forward	AACATCACCAATGCCACCAC
	Reverse	TTGCTCTCGGCTTCACTTTC
***LC3***	Forward	GCCTTCTTCCTGCTGGTGAA
	Reverse	TCCTGCTCGTAGATGTCCGC
***BECN1***	Forward	TCCATGCTCTGGCCAATAAG
	Reverse	ACGGCAGCTCCTTAGATTTG
***NEF2L2***	Forward	CCTCAAAGCACCGTCCTCAG
	Reverse	GCTCATGCTCCTTCTGTCGT
***HO-1***	Forward	CAAGCGCTATGTTCAGCGAC
	Reverse	GCTTGAACTTGGTGGCACTG
***β-actin***	Forward	GCAGGAGTACGATGAGTCCG
	Reverse	TGTCACCTTCACCGTTCCA G

### Enzyme-linked immunosorbent assay

The current study performed enzyme-linked immunosorbent assay analysis to determine the levels of proteins involved in inflammation, oxidative stress, apoptosis, and autophagy. In this regard, available ELISA kits designed to determine the rat levels of HO-1 (Cat:ab279414), IL-6 (Cat:ab234570), IL-1β (Cat: 255,730, Abcam Inc., Cambridge, United Kingdom), BCL-2 (Cat: E-EL-R0096), CASP9 (Cat: E-EL-R0163, Elabscience, Texas, USA), BAX (Cat: MBS935667), CASP3 (Cat: MBS261814), mTOR, LC3B (Cat: MBS9428940), BECN1 (Cat: MBS3808940), NFE2L2 (Cat: MBS012148, MyBioSource, Inc., San Diego, United States), NO (Cat:orb511103) and TNF-α (Cat: BMS622, Thermo Fisher Scientific Inc., Massachusetts, United States) were prepared. The measurement of levels was performed according to manufacturer protocols.

### Tissue preparation and total protein content measurement

Removed lung tissues were homogenized in an electrical homogenizer with ice-cold phosphate buffer, pH = 7.4, supplemented with antiprotease to obtain 1:10 (w/v) homogenate. Tissue homogenates were centrifuged at 4 ͦC for 15 min at 10,000 G to obtain supernatant. The supernatant was aliquoted and kept at -20 ͦC for further analysis.

The total protein was measured based on the Lowry assay with a few modifications [25]. In this regard, 0.5 ml of homogenate sample was mixed with Lowry solution, vortexed briefly, and then incubated for 20 min at room temperature at dark. Subsequently, 0.1 ml Folin (British Drug House, BDH) solution was added and vortexed briefly. After once more dark incubation for 45 min, the samples were vortexed briefly and the absorbance was recorded at 750 nm. The concentration was measured using the Bovine Serum Albumin (Sigma Aldrich, Canada Co) standard curve.

### The analysis of oxidative stress markers

In addition to previous steps, the current study aimed to assess the state of oxidative stress markers including superoxide dismutase (SOD), catalase (CAT), glutathione (GSH), glutathione peroxidase (GPx), malondialdehyde (MDA), and total antioxidant capacity (TAC). In this regard, the activity of SOD (Cat: NRZP-1122-ZP54), GPx (Cat: NK1120FY109), and CAT (Cat: NK1120FY048) enzymes, as well as the level of GSH (Cat: NK1120FY106), MDA (Cat: NK1120FY133), and TAC (Cat: NK1120FY169, Creative Biolabs, Newyork, USA) in tissue homogenates were measured using commercially available kits and according to the manufacturer’s protocol. The obtained values were normalized based on tissue protein content.

### Statistical analysis

Obtained data are presented as the mean ± SD. The statistical significance was determined using one-way ANOVA followed by the Tu-Key posthoc test with SPSS version 24.0 (IBM, Chicago, IL, USA). Moreover, GraphPad Prism version 8 (GraphPad Software, San Diego, CA, USA) was used for preparing graphics. The difference level of significance was set at *p*-value < 0.05.

## Results

### EGCG ameliorated LPS-induced alterations in the lung’s wet/dry weight ratio

**Fig. 1:**
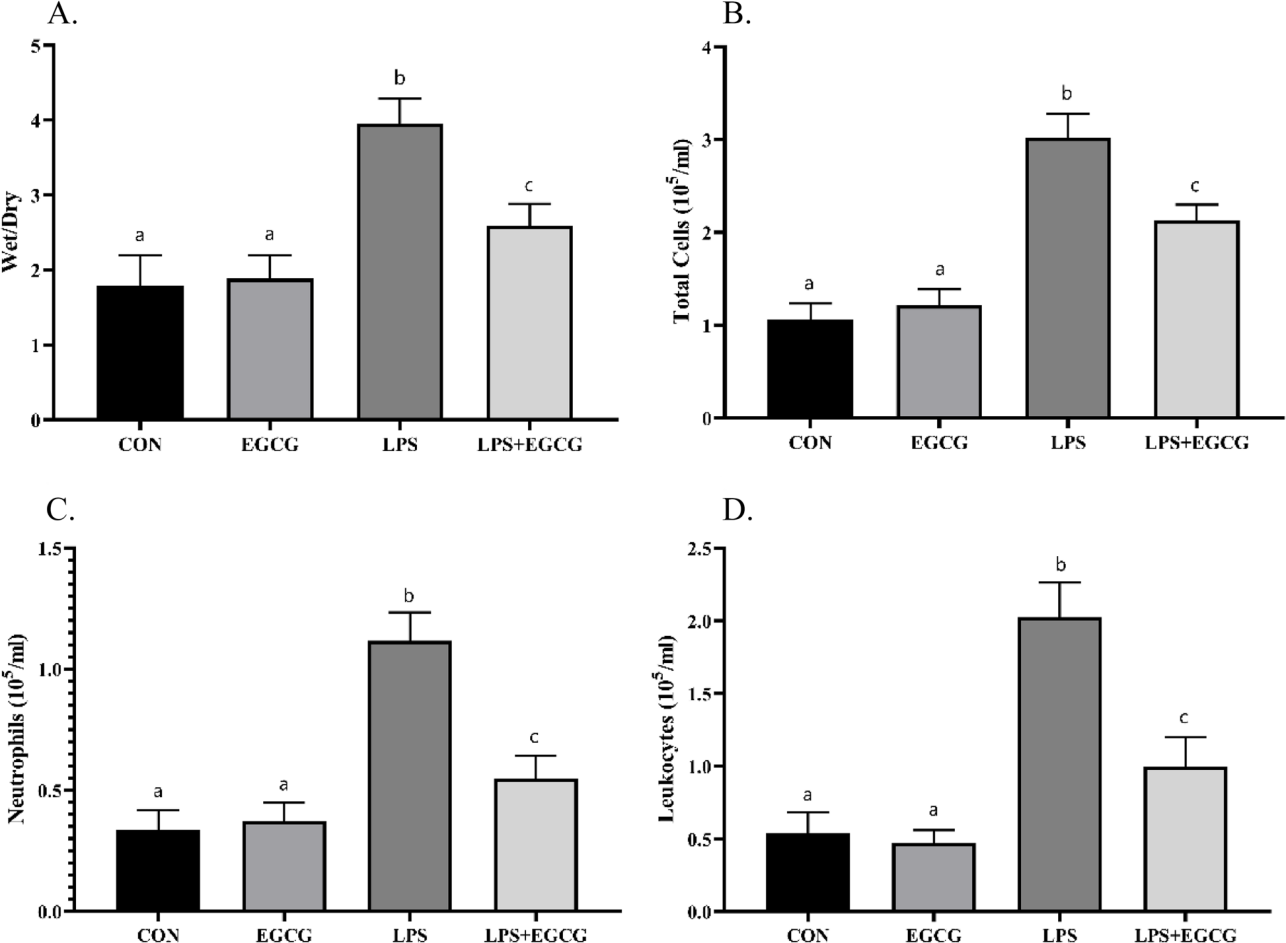
Wet/dry lung tissue weight ratio and number of cells in the BALF. LPS caused a significant increase in wet/dry weight ratio (**A**), total cell count (**B**), neutrophil count (**C**), and leukocyte count (**D**). Although the administration of LPS + EGCG caused a significant decrease in these parameters compared to the animal model of pneumonia, it also showed a significant difference with the control group. The lowercase letters presented on the bars describe the result of the statistical analysis, where different letters indicate significant differences between groups; the *p*-value < 0.05 was considered significant

Wet and dry weights of lung tissues were measured to obtain the W/D ratio. The findings of the present investigation demonstrated that the studied groups were significantly different in terms of wet/dry weight ratio (Fig. 1). In this regard, the wet/dry ratio in the CON and EGCG groups was 1.792 and 1.885, respectively (*p*-value = 0.972). Whereas, the treatment with LPS caused an increase of 120.65% compared to the CON group. Rats that were treated with LPS + EGCG showed significant differences with both CON and LPS groups (*p*-value < 0.0001).

### EGCG ameliorated LPS-induced inflammation in lung tissue

Counting cells in the BALF is one of the recommended ways to investigate inflammation in the lung tissue. The findings revealed that LPS increased the total number of cells in BALF by 2.8 times compared to the CON group (*p*-value < 0.0001). However, treatment LPS + EGCG caused an increase and decrease of 99.1% and − 29.10% compared to the CON and LPS groups, respectively (Fig. 1B, D. Also, the number of neutrophils and lymphocytes in the rats that were treated with LPS was 3.32 and 3.77 times higher than the CON group (Fig. 1C and D). Comparing the number of neutrophils and lymphocytes in the rats treated with LPS + EGCG showed a significant decrease compared to the LPS group (*p*-value < 0.0001). Similarly, a significant difference was obtained compared to the CON animals (*p*-value < 0.001).

**Fig. 2:**
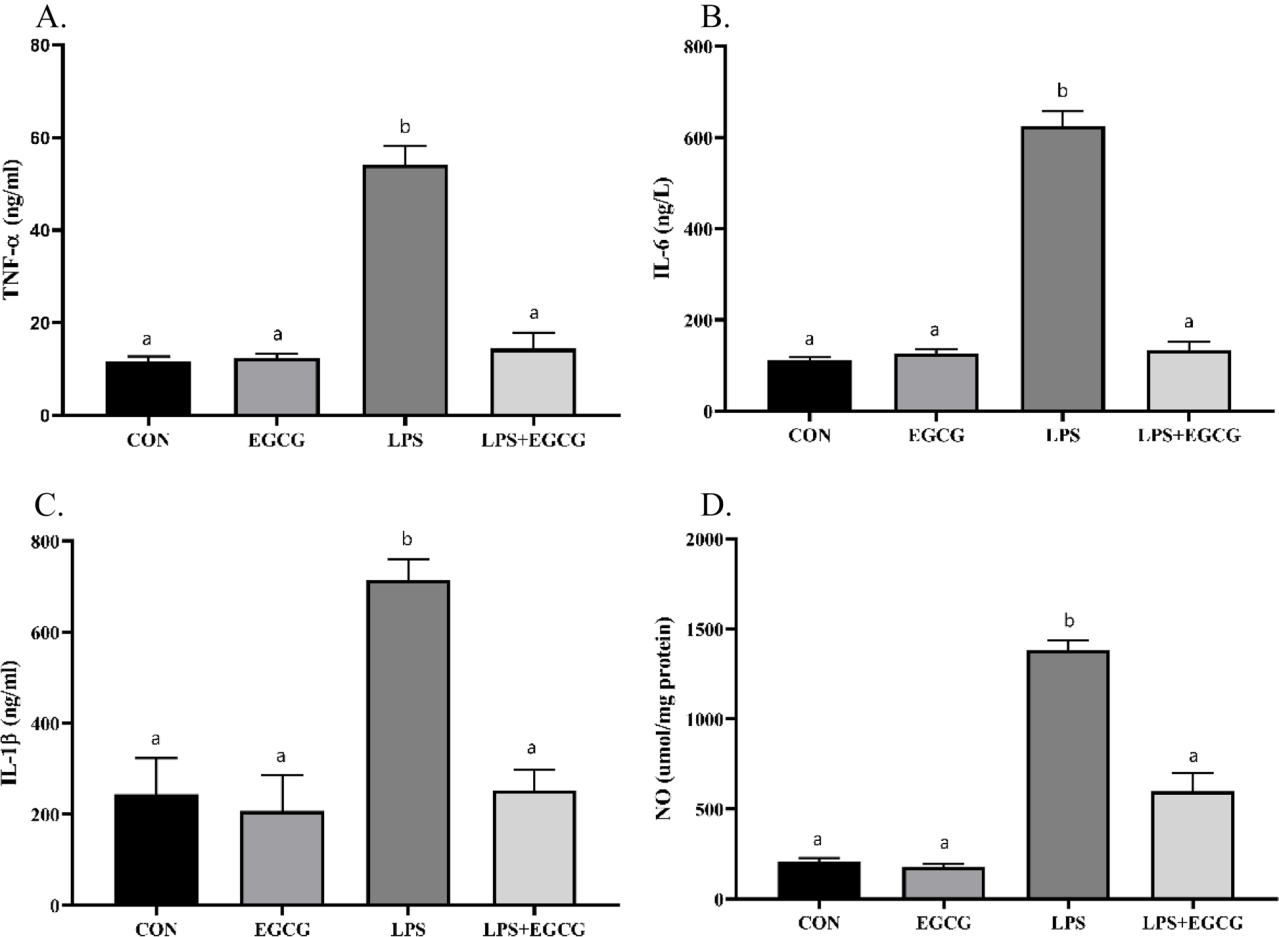
EGCG attenuated LPS-induced inflammation in the lung tissue. Animal models of pneumonia characterized high levels of inflammatory mediators including TNF-α (**A**), IL-6 (**B**), IL-1β (**C**), and NO (**D**). Administration of EGCG could significantly reduce the level of the mentioned factors. The lowercase letters presented on the bars describe the result of the statistical analysis, where different letters indicate significant differences between groups; the *p*-value < 0.05 was considered significant

In addition, the current study aimed to evaluate the state of inflammatory markers in the lung tissue by measuring the levels of TNF-α, IL-6, and IL-1β via available ELISA kits. The findings revealed that the levels of TNF-α, IL-6, and IL-1β in animals treated with LPS were approximately 4.6, 4.9, and 3.4 times more than CON animals (Fig. 2A, B, and C). Also, LPS + EGCG was able to significantly reduce the levels of TNF-α (14.4 vs. 59.1 ng/ml), IL-6 (133.5 vs. 623.8 ng/ml), and IL-1β (253.1 vs. 713.8 ng/ml) markers compared to the LPS group. No significant differences were found in the comparison of EGCG and LPS + EGCG groups with the CON (*p*-value > 0.05). Furthermore, LPS caused a significant increase in the level of NO compared to the CON group, although the combined administration of EGCG with LPS caused a significant decrease compared to LPS-treated animals (*p*-value < 0.0001).

### EGCG reduced LPS-induced apoptosis in lung tissue

**Fig. 3:**
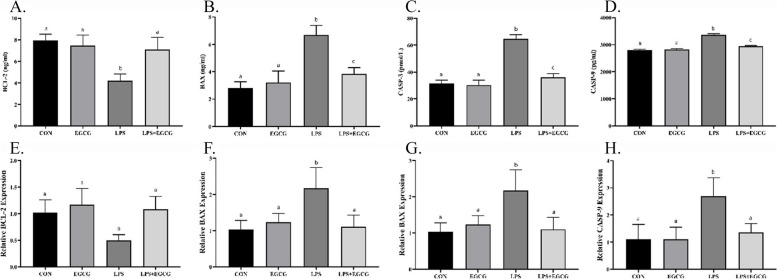
The suppression of LPS-induced apoptosis by EGCG. Apoptotic markers were measured at the protein level (**A**-**D**) and gene expression (**E**-**H**) through ELISA and RT-qPCR methods, respectively. EGCG caused the suppression of cell death induced by LPS through a significant increase in the level of BCL-2 anti-apoptotic factor (**A**, **E**) and a decrease in apoptotic mediators including BAX (**B**, **F**), CASP-3 (**C**, **G**), and CASP-9 (**D**, **H**). The lowercase letters presented on the bars describe the result of the statistical analysis, where different letters indicate significant differences between groups; the *p*-value < 0.05 was considered significant

To investigate apoptosis levels in lung tissue, the present study used RT-qPCR and ELISA methods to measure gene expression and levels of BCL-2, BAX, CASP-3, and CASP-9 proteins (Fig. 3). BCL-2 gene expression and protein level after treatment with LPS caused a significant decrease compared to the CON group (*p*-value < 0.001). This is even though LPS + EGCG caused a significant increase in the BCL-2 gene expression and protein level compared to the LPS group (*p*-value < 0.0001). However, the EGCG and LPS + EGCG groups did not demonstrate any significant difference from the CON group (*p*-value > 0.05).

On the contrary, LPS significantly increased the expression of proteins BAX (139.3%), CASP-3 (106.25%), and CASP-9 (19.90%) compared to the CON group (*p*-value < 0.0001). Also, investigating the expression of the mentioned genes showed a significant difference when groups LPS and CON were compared (*p*-value < 0.05). Moreover, the levels of BAX, CASP-3, and CASP-9 proteins were significantly different in LPS + EGCG animals compared to the CON group (*p*-value < 0.001).

### EGCG promotes autophagy in LPS-treated animals

**Fig. 4:**
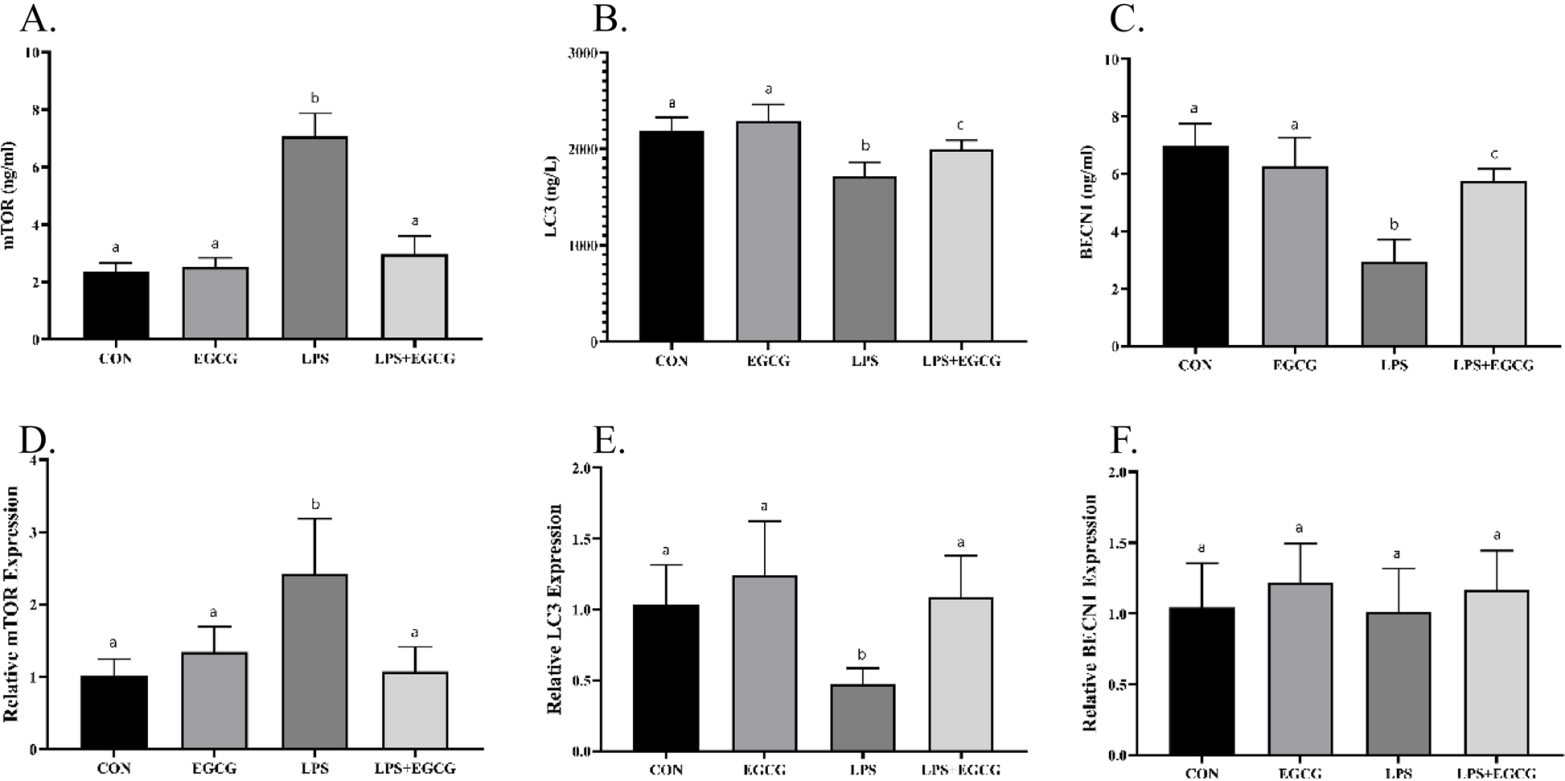
EGCG promoted autophagy in LPS-induced pneumonia. ELISA and RT-qPCR methods were used to measure the level of proteins (**A**-**C**) and the expression of genes (**D**-**F**) involved in autophagy, respectively. EGCG increased autophagy in lung tissue by decreasing the level of mTOR (**A**, **D**) and significantly increasing the level of LC3 (**B**, **E**) and BECN1 (**C**, **F**). The lowercase letters presented on the bars describe the result of the statistical analysis, where different letters indicate significant differences between groups; the *p*-value < 0.05 was considered significant

The investigation of autophagy markers, including mTOR, LC3, and BECN1 was followed at the level of the gene (using RT-qPCR method) and protein (using ELISA method) expression to measure the changes caused by the administrated compounds on the autophagic flux in the lung tissue (Fig. 4). The findings revealed that LPS significantly increased the level of mTOR protein by 197.9% compared to CON, while the level of LC3 and BECN1 proteins remarkably decreased by 21.39% and 57.80%, respectively. In addition, the expression of *mTOR* and *LC3* encoding genes showed a significant difference between the LPS and CON groups (*p*-value < 0.001), however, no significant difference was revealed regarding *BECN1* gene expression between LPS and CON (*p*-value > 0.05).

Although the administration of EGCG combined with LPS caused a significant improvement in mTOR, LC3, and BECN1 protein levels compared to the LPS group (*p*-value < 0.001), a significant difference was also obtained in terms of LC3 and BECN1 levels when compared to the CON animals. Nevertheless, the expression of *mTOR* and *LC3* genes in the LPS + EGCG treated animals showed a significant difference only with the LPS group (*p*-value < 0.05).

### EGCG attenuated LPS-induced oxidative stress

**Fig. 5:**
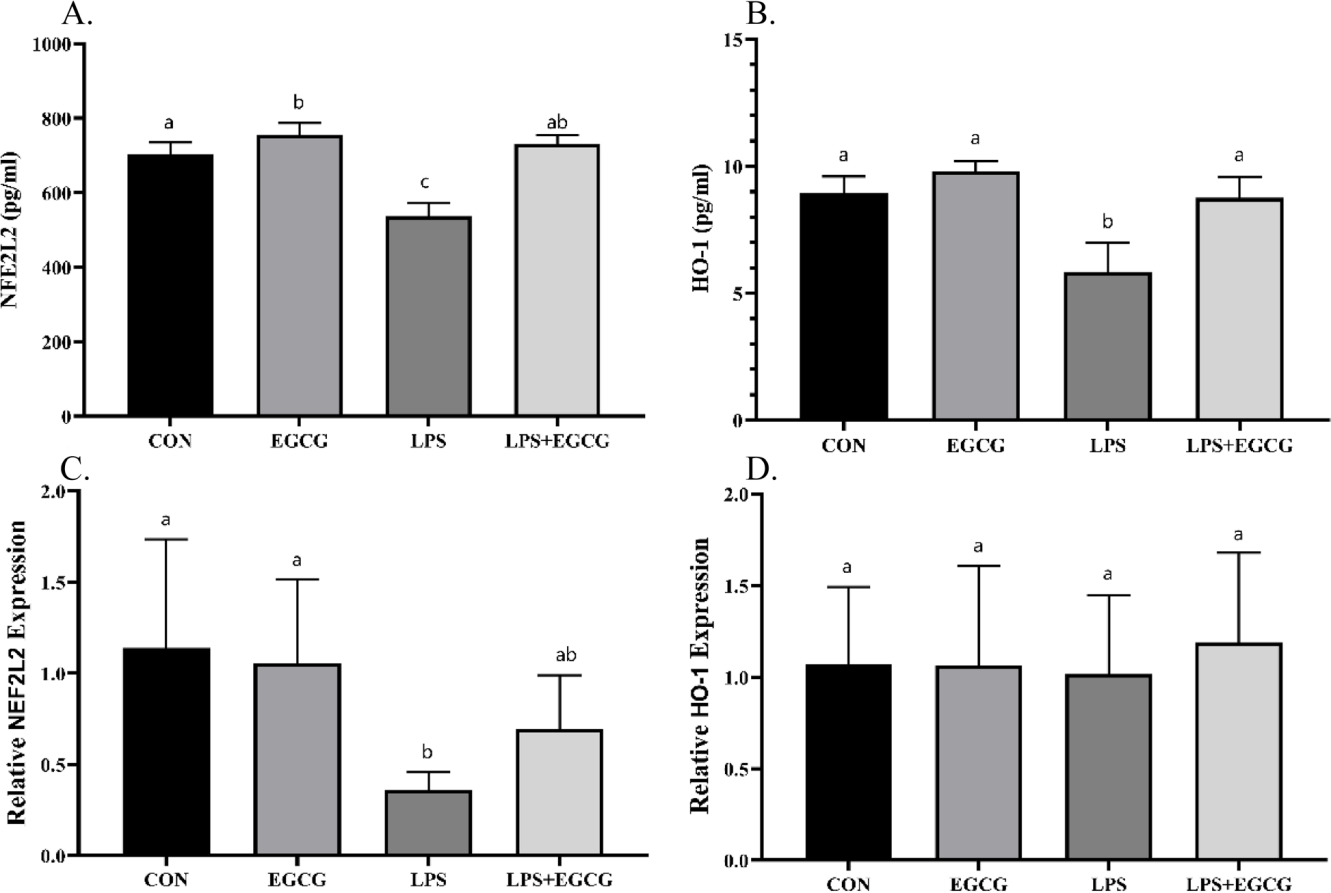
The overexpression of antioxidative regulators by EGCG. The level of NFE2L2 (**A**) and HO-1 (**B**) proteins along with the expression of encoding genes (**C** and **D**, respectively) were measured to assess the state of upstream regulators of oxidative metabolism. It appears that in the animal model of pneumonia, the level of NFE2L2 and HO-1 decreased significantly, while the administration of EGCG caused a significant upregulation. The lowercase letters presented on the bars describe the result of the statistical analysis, where different letters indicate significant differences between groups; the *p*-value < 0.05 was considered significant

The assessment of stress status was performed by measuring the levels of *NFE2L2* and *HO-1* gene expression via the RT-qPCR method as well as using available calorimetric kits to measure SOD, GPx, and CAT activity and GSH, MDA, and TAC levels. Several studies have suggested NFE2L2 and HO-1 as regulators of oxidative balance, which are able to respond to the overproduction of free radicals by changing the level of antioxidant defenses [26]. The present study investigated the level of these two markers using RT-qPCR and ELISA methods (Fig. 5). The findings revealed that LPS caused a significant decrease in NFE2L2 and HO-1 proteins in the lung tissue compared to the CON group. Moreover, the expression of *NFE2L2* and *HO-1* genes in the LPS group was significantly reduced when compared to the CON (*p*-value < 0.0001). Administration of EGCG in LPS-treated animals increased the NFE2L2 protein level by 35.79% compared to the LPS group (*p*-value < 0.0001), although no significant difference was found between the LPS + EGCG and CON group. Moreover, the level of HO-1 protein in the LPS + EGCG group was significantly increased compared to LPS (*p*-value < 0.001), while no significant difference compared to the CON was obtained (*p*-value > 0.05). Although the *NFE2L2* gene expression in the LPS group had a significant decrease of 68.57% compared to the CON, no significant difference was obtained between the LPS + EGCG and LPS groups. *HO-1* gene expression did not determine any significant difference between the studied groups (*p*-value > 0.05).

In addition to determining the protein and the gene expression level of NFE2L2 and HO-1 genes, the present study measured the activity of several enzymes involved in the response to oxidants as well as markers of oxidative stress (Table 2). The findings revealed that the activity of SOD, CAT, and GPx enzymes in the LPS group had a significant decrease compared to the CON (*p*-value < 0.001). In addition, the administration of LPS caused a significant decrease in the levels of GSH and TAC compared to the CON. On the contrary, a significant increase in the level of MDA was found when the LPS and CON groups were compared (*p*-value < 0.0001). Interestingly, administration of EGCG combined with LPS caused a significant difference with the LPS group (*p*-value < 0.001).

**Table 2:** The analysis of oxidative status in the lung tissue

Markers	CON	EGCG	LPS	LPS + EGCG	*P*-value
***SOD*** U/mg protein	6.63 ± 0.54^a^	7.52 ± 0.29^a^	4.20 ± 1.01^b^	6.46 ± 1.03^a^	< 0.0001
***CAT*** mU/mg protein	3.74 ± 0.23^a^	3.69 ± 0.21^a^	2.48 ± 0.27^b^	3.06 ± 0.57^a^	< 0.001
***GPx*** mU/mg protein	51.36 ± 1.86^a^	46.79 ± 2.15^a^	28.09 ± 2.43^b^	35.47 ± 4.35^c^	< 0.0001
***GSH*** nmol/mg protein	1.59 ± 0.29^a^	1.29 ± 0.24^a^	0.34 ± 0.29^b^	1.13 ± 0.31^c^	< 0.0001
***TAC*** µmol Fe2+/mg protein	2.40 ± 0.18^a^	3.02 ± 0.28^b^	1.55 ± 0.23^c^	2.52 ± 0.22^a^	< 0.0001
***MDA*** µmol/mg protein	0.56 ± 0.26^a^	0.53 ± 0.38^a^	2.68 ± 0.27^b^	1.54 ± 0.36^c^	< 0.0001

The suppression of antioxidative enzymes, reduction in GSH and TAC levels, and the increment in MDA levels revealed the induction of oxidative stress in rats with pneumonia. However, the administration of EGCG in animal models of pneumonia significantly ameliorated oxidative stress. Different letters represent significant differences; the *p*-value < 0.05 was considered significant

## Discussion

Pneumonia is considered a major disease with high prevalence rates threatening human life [27, 28]. Previous studies have assumed dysregulation of molecular pathways such as apoptosis and autophagy [5] along with the induction of inflammation and oxidative stress [8] as the main underlying mechanisms involved in disease progression. However, ongoing investigations are being conducted to provide novel therapeutic strategies. Phytochemicals are among the compounds that have been proposed to treat pneumonia due to their ability to regulate apoptosis and autophagy, as well as representing antioxidant and anti-inflammatory properties [29, 30]. The present study aimed to evaluate the ability of EGCG to improve dysregulated molecular pathways in an animal model of LPS-induced pneumonia.

The present findings demonstrated that EGCG was able to improve lung tissue damage in an animal model of pneumonia. In addition, the increase in the number of total cells, neutrophils, and leukocytes in BALF induced by pneumonia was ameliorated by EGCG. An increase in the number of white blood cells is considered one of the indices of the immune system’s response to stimuli, which is often associated with the release of inflammatory cytokines [31, 32]. Although the increase in the level of inflammatory cytokines is an indicator of the response to pathogens, which occurs to confront the invading agent, it may result in tissue damage [33]. The findings revealed that EGCG can significantly reduce the increased levels of TNF-α, IL-6, and IL-1β induced by LPS. Concordantly, several previous studies have hypothesized the ability of phytochemicals to attenuate the inflammation caused by pneumonia attributed to herbs’ anti-inflammatory properties as well as their ability to regulate the signaling pathways modulating inflammatory responses [34–36].

Along with inducing inflammation, pneumonia threatens cell survival and induces cell death which may contribute to intensifying lung damage [37, 38]. The findings of the present study revealed that in the animal model of pneumonia, the level of BCL-2 was significantly reduced, while other apoptotic markers including BAX, CASP-3, and CASP-9 were remarkably increased. BCL-2 is described as the anti-apoptotic member of the BCL-2 family that prevents apoptosis either by sequestering proforms of CASPs, known as death-driving cysteine proteases, or by preventing the release of mitochondrial apoptogenic factors [39]. Whereas other factors are considered to be apoptosis developers. Indeed, BAX translocates to the mitochondrial membrane upon pro-apoptotic insult, forms the apoptotic pore within the membrane, and finally triggers activation of the CASP cascade, all of which contribute to apoptotic cell death [40, 41]. Interestingly, the present results showed that EGCG, through the modulation of BCL-2, BAX, CASP-3, and CASP-9, prevented pneumonia-induced apoptosis. There is a plethora of evidence suggesting the anti-apoptotic properties of phytochemicals such as quercetin [42], resveratrol [43], and curcumin [44] that improve pneumonia. In fact, preventing the chronic exacerbation of inflammatory responses, suppressing apoptosis, and preventing the induction of oxidative stress (discussed further) by affecting upstream signaling pathways are the main mechanisms by which phytochemicals ameliorate pneumonia [45].

In addition, the findings of the present investigation determined that in the pneumonia model of animals, the level of mTOR in lung tissue was significantly increased, while a considerable decrease in the levels of LC3 and BECN1 was induced after pneumonia. These results indicate the suppression of autophagy caused by pneumonia in lung tissue because mTOR is known as a tight upstream suppressor of autophagy (via phosphorylation-dependent inhibition of ULK1/2), while LC3 and BECN1 are pivotally involved in the formation of the autophagosome and its fusion with lysosomes [46, 47]. Autophagy plays a dual approach in cell survival, as it provides cell-required energy and increases cell life through the removal of inefficient organelles and macromolecules, and contradictory, as a mechanism of programmed cell death may determine the fate of cells [47, 48]. Several studies have documented that autophagy plays a crucial role in suppressing bacterial and viral infections, therefore autophagy suppression in pneumonia may be a necessary mechanism for disease progression [49–51]. Importantly, EGCG was able to modulate the levels of mTOR, LC3, and BECN1 and thereby may be involved in the promotion of autophagic flux. Accordingly, previous studies have attributed the therapeutic effects of phytochemicals in improving pneumonia to the ability of these compounds to promote autophagy [52, 53].

Alternation of oxidative metabolism is one of the leading pathogenic mechanisms involved in the development and progression of pneumonia. Indeed, it has been suggested that oxidative stress increases and antioxidant activities diminish in children with acute pneumonia [54]. In addition, oxidative stress is considered one of the most important mechanisms by which asthma exacerbates vascular dysfunction in pneumonia [8, 55]. The findings of this study showed that EGCG was able to potentially suppress oxidative stress induced by pneumonia through increasing antioxidant activities. The anti-oxidative ability of phytochemicals has been documented as the main characteristic of these compounds, which makes them promising candidates for a wide range of therapeutic strategies from the treatment of cancer and chronic diseases to confronting infections and reducing toxicity [24, 56–58].

Importantly, the current results have shown the ameliorative properties of EGCG on lung damage caused by LPS-induced pneumonia in rat models. Nevertheless, EGCG cannot be considered a substitute for current treatment strategies, only may suggest it as a complementary treatment along with common treatment options. In addition, conducting further studies, especially similar animal studies and clinical trials, appears to be necessary to validate the findings of the present study and determine the appropriate dosage to use. Therefore, the obtained results can be considered a basis for further studies to reveal how EGCG affects the activity of immune cells, preserves the histoarchitecture and physiological function of the lung tissue, and modifies intracellular and/or extracellular mechanisms to confront pneumonia.

## Conclusion

Pneumonia treatment is facing significant challenges and providing novel strategies, especially complementary therapeutic options, is followed in continuous studies. The present study aimed to measure the effects of EGCG on the changes in markers of apoptosis, autophagy, inflammation, and stress in a rat model of LPS-induced pneumonia. The findings of the present study revealed that EGCG alleviated the LPS-induced destructive alterations in a rat model of pneumonia via suppressing inflammation, apoptosis, and oxidative stress as well as inducing autophagy. These results may indicate the promising properties of EGCG as a novel complementary strategy for pneumonia management, although further studies, in particular clinical trials, are encouraged in this regard.

## Data Availability

The data that support the findings of this study are not openly available due to reasons of sensitivity and are available from the corresponding author upon reasonable request.
